# Emerging Function of Ecotype-Specific Splicing in the Recruitment of Commensal Microbiome

**DOI:** 10.3390/ijms23094860

**Published:** 2022-04-27

**Authors:** Yue-Han Li, Yuan-You Yang, Zhi-Gang Wang, Zhuo Chen

**Affiliations:** 1 State Key Laboratory Breeding Base of Green Pesticide and Agricultural Bioengineering, Key Laboratory of Green Pesticide and Agricultural Bioengineering, Ministry of Education, Research and Development Center for Fine Chemicals, Guizhou University, Guiyang 550025, China; leeenxiuu@163.com (Y.-H.L.); gs.yangyy16@gzu.edu.cn (Y.-Y.Y.); 2 School of Life Science and Agriculture Forestry, Qiqihar University, Qiqihar 161006, China; 3 Heilongjiang Provincial Technology Innovation Center of Agromicrobial Preparation Industrialization, Qiqihar 161006, China

**Keywords:** alternative splicing, plant genotype, plant–microbe interaction, microbiome assembly, post-transcriptional regulation, proteogenomics

## Abstract

In recent years, host–microbiome interactions in both animals and plants has emerged as a novel research area for studying the relationship between host organisms and their commensal microbial communities. The fitness advantages of this mutualistic interaction can be found in both plant hosts and their associated microbiome, however, the driving forces mediating this beneficial interaction are poorly understood. Alternative splicing (AS), a pivotal post-transcriptional mechanism, has been demonstrated to play a crucial role in plant development and stress responses among diverse plant ecotypes. This natural variation of plants also has an impact on their commensal microbiome. In this article, we review the current progress of plant natural variation on their microbiome community, and discuss knowledge gaps between AS regulation of plants in response to their intimately related microbiota. Through the impact of this article, an avenue could be established to study the biological mechanism of naturally varied splicing isoforms on plant-associated microbiome assembly.

## 1. Introduction

The interaction between host plants and their commensal microbiome has become a research hotspot in recent years. As sessile organisms, plants have evolved a series of mechanisms to cope with changing environments [1,2]. In fact, the interaction between plant immunity and the plant microbiota is bidirectional, with plants, microbiota, and the environment forming a complex system and working together to coordinate the plant microbiota. An increasing number of researchers have suggested that those plant-associated microorganisms can be studied together with their host plants as one combined holobiont in the field [3]. Benefits from adequate mutualistic interactions will improve plant growth and soil productivity, inhibit pathogen propagation, enhance plant tolerance to adverse environments, and accelerate nutrient recycling in fields [4], ultimately providing an effective way for agriculture to increase crop yield in a sustainable manner [5].

Influencing factors that affect this mutualistic interaction have been extensively reported, including internal and external factors in both plants and microorganisms [6]. Specifically, there is a growing body of evidence that plant genetics information is crucial in response to microbial colonization, extending the knowledge of plant biology to other disciplines such as microbiology and ecology [7]. Studies using different plant ecotypes or cultivars have been conducted to unravel the underlying mechanism of this interaction [3]. For example, plants decode genetic information to determine the composition of root exudates [8], root architecture [9], or ingredients for the microbiome assembly to respond at its surface or rhizosphere. Current reports aim to understand the diversity and dynamics of indigenous microbial communities coupled with the identification of core microbiota or keystone species [3]. However, most of the studies are descriptive, paying little attention to the underlying mechanism to mediate this interaction [1,3].

In addition, plants can translate their genetic differences by adjusting the repertoire of proteins they express in response to surrounding abiotic and biotic factors. One post-transcriptional regulatory mechanism, notably alternative splicing (AS), has emerged as a pivotal process for plants, to adapt to changing environments by significantly increasing proteome diversity or fine-tuning transcript abundance [10]. To date, approximately 95% of human intron-containing genes undergo AS regulation and up to 35% of human diseases are related to mutations that can affect splicing [11,12]. Importantly, single nucleotide polymorphisms (SNPs) observed at a splice site will affect the expression of certain transcript isoforms, thus resulting in phenotypic diversity in research on human diseases [13]. In plants, 60–83% of intron-containing genes undergo AS [14]. Natural variation of AS, identified as splicing quantitative trait loci (functions of sQTLs) [15], and its contribution to plant traits has been observed by using population-level transcriptome analyses, providing evidence for linking genetic variants to plant trait variation [16,17,18]. However, which function of sQTLs contribute to plant–microbe interactions is poorly understood. To this end, we first introduce the current progress of these research questions in this review article. Then, we discuss the possible mechanism to connecting diverse plant ecotype–phenotype linkages, splicing isoforms, and commensal microbiomes. In addition, we discuss further investigation using modern high-throughput omics technology to identify target plant alleles that can modulate interactions between host plants and their intimately related microbiota.

## 2. Ecotype-Specific Microbial Community in Plants

### 2.1. Host Maintanence of Commensal Microbiome

The microbial community is a crucial environmental component, as approximately 10 billion microorganisms are present in every gram of soil [19]. The interaction between plants and microorganism communities is a vital driver of ecosystem functioning [20] and can affect the maintenance of agricultural ecosystems [21]. These microorganisms will form complex co-associations with plants and have necessary roles in promoting the productivity and health of the plant type in natural environments. In particular, plant-associated microbiota and plants from a ‘holobiont’, and evolutionary selection among microbes and plants contributes to the steadiness of an ecosystem [22,23]. Host-associated microbial communities have a crucial role in shaping the health and fitness of plants [24]. Diverse microbial communities colonize plant surfaces and tissues, and beneficial microbial groups provide plants with a large array of life supporting functions such as resilience to organic phenomena and abiotic stresses, growth promotion, and nutrient acquisition [25,26]. It is well known that many beneficial microorganisms in the soil after planting plants will enrich the roots of the plants [27]. Plants and microorganisms work together as an important part of plant growth and illustrate the importance of plant hosts; however, there is a need to better understand the governing plant–microorganism interactions at the community level and explore their potential agricultural application value. It was found that the composition of microbial communities among different plants is diverse in bulk soils under different vegetation covers [28]. Furthermore, the field bacterial community structure in the bulk and inter-root soils with different plant diversity treatments was examined, and significant differences in bacterial communities were observed in both bulk and inter-root soils [29]. In recent years, culture-independent high-throughput sequencing has greatly expanded the repertoire of microorganisms known to reside in and on plants [30,31,32]. Plant growth-promoting rhizobacteria (PGPR) are gradually attracting public research attention. If we visualize an ideal agricultural picture, crops produced should be equipped with disease resistance, salt tolerance, drought tolerance, serious metal stress tolerance, and higher nutritional value. One way is to use soil microorganisms (bacteria, fungi, algae, etc.) to achieve this goal [33]. Soil microbial populations are immersed in a framework of interactions known to have an effect on plant fitness and soil quality. Cooperative microbial activities may be exploited as a low-input biotechnology to assist sustainable, environmentally friendly, agro-technological practices. Among these potential soil microorganisms, PGPR bacteria are the most promising research targets. PGPR could also be used to enhance plant health and promote the plant growth rate without environmental contamination [34]. There have also been deeper developments in recent years regarding the separation of PGPR, which also has a positive effect on plants. The most remarkably studied bacteria in relation to biocontrol are members of the genera *Pseudomonas* spp., *Bacillus* spp., *Azospirillum* spp., and *Streptomyce* spp. [35,36,37]. For example, *Bacillus thuringiensis* is renowned as a good bioinsecticidal bacterium, and acyl homoserine lactone lactonase created by *B. thuringiensis* can open the lactone ring of N-acyl homoserine lactone, a signature molecule within the bacterial quorum-sensing system [38]. The capacity of microorganisms to rescue plants’ capacity of stress has been recently studied [39,40,41]. For example, wheat microbiome bacteria can relieve plant stress, and a compound secreted by the bacteria (phenazine-1-carboxamide) directly affects the activity of fungal protein FgGcn5, a histone acetyltransferase. This results in deregulation of simple protein acetylation at H2BK11, H3K14, H3K18, and H3K27 in *F. graminearum*, as suppression of fungal growth, virulence, and mycotoxin biogenesis [41]. Therefore, the associated degree of antagonistic bacteria can inhibit the growth and virulence of a plant pathogenic fungus by manipulating fungi simple protein modification. Strains of *Chitinophaga* spp., *Chryseobacterium* spp., *Flavobacterium* spp., *Microbacterium* spp., *Pseudomonas* spp., *Sphingomonas* spp., *Stenotrophomonas* spp., and *Xanthomonas* spp. are typically found to complement plant responses to totally different pathogen/pest attacks. However, the benefits of microorganisms on plants may occur via two modes. For example, a change in the structure of the inter-root microbiome would give the common tomato the property of resistance to wilt. The model dicot plant *Arabidopsis thaliana* specifically promotes three bacterial species within the rhizosphere upon foliar defence activation by the mildew pathogen *Hyaloperonospora arabidopsidis* [42]. 2,4-diacetylphloroglucinol (Phl) produced by *Pseudomonas* on roots of wheat grown in a soil suppressive to take-all of wheat. Phl-producing fluorescent *Pseudomonas* are key elements of the natural biological control that operates in take-all-suppressive soils [43]. Recruitment of microbes, via plant hosts, could have evolved to prime plant defence communication pathways and inhibit the expansion and virulence of pathogens that consequently ameliorate plant stresses [44,45,46]. This suggests that plant hosts largely determine the changes in plant microbial communities. Therefore, we believe that plants depend on microorganisms to bring them positive effects, and they have unique ways to ‘communicate’. These works need to be explored continuously and need more research to explore important research implications.

### 2.2. Function of Microbiome in Plant Defense and Immune Systems, Corollary of the Relationship between Plant Active and Passive Immunity

The plant microbiome is not static: its structure and therefore the provided host functions in response to stresses and environmental stimuli [47,48,49,50]. Recent studies suggest that the changes within the microbiome are not merely passive responses of plants, but rather a consequence of millions of years of coevolution [39,40,41,45,51].

Emerging evidence has indicated that plant-associated microbiomes are closely related to plant health [52] and the helpful features of plant-associated microbes will boost the immune responses in plants against biotic/abiotic environmental constraints [53,54]. In high abundance, beneficial microbes directly inhibit pathogens by producing antimicrobial compounds. However, beneficial microbes can also inhibit pathogens indirectly by stimulating the immune system of plants, a development referred to as induced systemic resistance (ISR) [55]. Two forms of systemic immunity triggered by plant–microbe interactions, systemic acquired resistance (SAR) and ISR, are classified due to differences in the site of induction and the manner in which the microbes are induced. Both SAR and ISR influence growth regulator crosstalk towards enhanced defence against pathogens, which affects the composition of the plant microbiome. ISR inducers include PGPR, such as *Pseudomonas* spp., *Bacillus* spp., *streptomyces* spp., and plant growth-promoting fungi (PGPF), as well as *Trichoderma* spp. and *Serendipita indica* (formerly *Piriformospora indica*) [56,57,58,59]. In plants, interaction with beneficial microbes of the microbiome will additionally result in the activation of the plant’s system, not least in the form of ISR. Reciprocally, the composition of the plant microbiome is influenced by plant immunity, which we call active immunity. This appears to be only determined by two main mechanisms thus far: direct microbe–microbe interactions and stimulation or priming of the plant immune system. For instance, a molecule secreted by the *Pseudomonas piscium* ZJU60 strain, which was isolated from infected wheat head, antagonizes the flora *Fusarium graminearum* by inhibiting its simple protein acetyltransferases [41]. The ability to antagonize other microbes, together with pathogens, may be a common attribute in bacteria isolated from the *Arabidopsis* leaf microbiome [60]. These studies all suggest that plant microbiota are a rich source of pathogen antagonists that act through direct inhibition [61].

Plant–pathogen interactions are mediated by the interplay of multifaceted processes, which are expedited by pathogen- and plant-oriented molecules [62,63]. Pathogens and commensal microbes that survive competition with different soil and plant-associated microbes then encounter the plant innate system, principally microbe-associated molecular patterns (MAMPs), triggered immunity (MTI), and effector triggered immunity (ETI), two layers of molecular defence referred to within the advanced zigzag defence model [64]. PTI and ETI both depend on SA, and each induce a systemic, SA-dependent defence response that is known as systemic acquired resistance (SAR) [65,66,67]. SAR may be a durable style of resistance against a broad spectrum of (hemi-)biotrophic pathogens [68].

The vital next step, currently, is to check these molecular mechanisms and unravel how commensal microbes move with plant immunity under various plant conditions. Such interactions between the system and microbiome are likely critical for plant defence against diverse stresses and influence host microbiota associations. Evidently, internal secretion signalling pathways of jasmonic acid (JA), salicylic acid (SA), and ethylene (ET) and their interactions are crucial in regulating plant defences and their associated microbiota [69,70]. Biotrophic and hemi-biotrophic pathogens that depend upon living host tissues and cells are fended off chiefly through SA-dependent immune mechanisms. Other findings show that the diversity of the endophytic microorganism community in *Arabidopsis* leaves is in response to the associated degree of activation of the SA signalling pathway [70] and SA signalling is involved in the modulation of root microbiota.

Taken together, unravelling the interactions between plant defence systems, microbiomes, and environmental factors will be an essential next step for understanding plant selection of microbes and immune modulation shifts of plant microbiota of various environmental conditions.

### 2.3. Plant Hosts Selectively Recruit Microbes via Ecotype Specificity

In light of growing concern over the threat of water and nutrient stress facing terrestrial ecosystems, particularly those used for agricultural production, increased emphasis has been placed on understanding how abiotic stress conditions influence the composition and functioning of the root microbiome and its ultimate consequences for plant health. However, under abiotic stress conditions, the composition of root microorganisms will change accordingly, which will be reflected in changes in root secretions. Although there are many conclusions about abiotic stresses on microbial community alteration, in recent years, attention has gradually shifted to the genotype differences of plant hosts. Following the appearance of next-generation sequencing, many studies have characterized the rhizospheric wheat microbiome and investigated the influence of the compartment (rhizoplane vs. endosphere), crop management, or wheat genotypes on the diversity and structure of those complicated microbial communities [71,72,73,74]. For many crops, studying soil microbial structure is indisputably the most necessary issue in crop management and plant genotype research. A number of studies have demonstrated that plants confirm changes of the microbial community under the same environmental conditions. There are many studies on the effect of different genotypes of the same plant on microorganisms (Table 1). The importance of genotype–environment interactions is in the structural assembly of plant microbiomes. For example, the reference plant *Arabidopsis thaliana* demonstrates that host cultivars (genotypes) mediate a weak but measurable impact on the root-associated microbiota [75]. Plant genotypes and soil have specific effects on the wheat rhizosphere microbial community [76]. The effect of plant genotypes on the inter-root microbial community of maize showed that the two genotypes had a significant effect on the inter-root microbial community of maize [77]. Lamit investigated different cottonwood genotypes supporting different aboveground fungal communities [78]. Jiang et al. investigated the inter-root bacterial community of 12 Rabbiteye blueberry (RB) cultivars and demonstrated that inter-rooting from plant cultivars affects the bacterial association network [79]. Microbial community (SMC) structure and root turnover were assessed in two contrasting *Lolium perenne* cultivars (AberDove and S23), and microbial communities with differences were discovered [80]. Hou et al. found significant differences in the composition of inter-root bacterial communities of different rice varieties under metal contamination conditions [81]. A significant genotypic variation in rhizosphere microbial communities in rice plants was reported [82]. Huang et al. showed significant differences in the inter-root microbial communities of two varieties of kale-type oilseed rape [83]. By analysing the soil samples of three different peony species, it was found that the microbial community structure was greatly influenced by the peony species [84]. Zhou et al. found significant differences in the inter-root microbial communities of four cucumber varieties [85]. A comparison of different genotypes of soybean [5], winter wheat [86], and maize [4] also revealed that the bacterial community structure differed among cultivars.

All of this evidence suggests that microbial communities change in response to changes in hosts, so a hypothesis has been proposed that plants will recruit different microorganisms by releasing different root secretions based on their own demand recruitment to relieve stress on their own growth or when they encounter stressful environments. Specifically, a mechanism employed by plants is the ‘cry for help’ strategy [87], a phenomenon whereby plants experiencing abiotic, pest-induced, or pathogen-induced stresses recruit helpful microbes/traits from the environment by employing a range of chemical stimuli to boost their capacity to combat stresses [88,89]. For example, some soils conditioned by take-all disease-infected wheat may lead to less take-all in future generations [43]. By studying the composition and metabolic potential of inter-root bacterial communities of different common bean (*Phaseolus vulgaris*) cultivars with different levels of resistance to the fungal root pathogen Fusarium (Fox), differences in microbial composition between susceptible and nonsusceptible cultivars and higher network complexity of resistant cultivars were found. Pseudomonadaceae and Bacillaceae had a high abundance in the rhizosphere of the Fox-resistant cultivar. Breeding for Fox resistance in common bean could have co-selected other unknown plant traits that support the next abundance of specific helpful microorganism families in the rhizosphere with functional traits that reinforce the primary line of defence [90]. Microbiome structures differed between the two tomato cultivars and transplantation of rhizosphere microbiota from resistant plants suppressed disease symptoms in susceptible plants. Comparative analyses of rhizosphere metagenomes from resistant and susceptible plants enabled the identification and assembly of a *flavobacterial* genome that was much more abundant in the resistant plant, which could suppress *R. solanacearum*-disease development in a susceptible plant [45]. Thus, on the far side of classical ‘adapt or migrate’ strategies, accumulating evidence suggests that plants use the ‘cry for help’ strategy as a full-of-life method that enables them to learn from microorganism associations under stresses. The host is the subject, and it is unclear in what way they recruit microorganisms; therefore, mining the mechanism of plant–microbe interactions will be an important part of future plant breeding. However, this theory still needs more evidence to prove it.

### 2.4. Emerging Factors That Are Responsible for Microbe Recruitment

It is not surprising that various active genes of the soil microbiota play important roles in competition or cooperation with other microbes. Microorganisms synthesize completely different products that affect microbe–microbe interactions. Distinct and numerous gene clusters for biosynthesis of natural products have been identified in plant-associated microorganisms [91,92]. Additionally, the organic chemistry diversity of root exudates was analysed for alterations within the presence or concentrations of metabolites (metabolomics) to determine how root exudation drives stress-induced microbiome assembly. The key metabolic compounds are isolated from the rhizosphere for potential and tested in vitro for interactions with plant microorganism symbionts and pathogens and, in turn, plant health. The addition of various exudate mixtures to plant monocultures increased microorganism diversity. Some studies also found that higher plant diversity was associated with higher microbial diversity. Major challenges remain, such as analysis of root exudation in natural settings, mostly due to the chemical quality of various soil types. A few plant products that affect the rhizosphere microbiome are known. For example, 2,4-diacetylphloroglucinol [50], peroxidases and oxylipins [93], benzoxazinoids [94], phenylpropanoids [95], flavonoids [93], coumarins [96], triterpenes, mucilage [97], and aromatic compounds [98] are reported to attract microbes that benefit plant defence and nutrition. However, how these compounds are synthesized and how they regulate the microbe interplay, along with their underlying biochemical mechanisms, remains to be explored. Although each plant produces exudates, the number and composition of root exudates vary. First, exudation is outlined by the genotype of the host. Nineteen *Arabidopsis* root metabolic patterns and their variability between plants and naturally occurring germplasm were analysed, and for some secondary metabolites, the ratio of total plant-to-plant variability was high and significant [99]. Exudation is modulated by abiotic stresses: the amounts of exuded amino acids, sugars, and organic acids modified in maize fully grown in phosphate-, iron-, nitrogen-, or potassium-deficient conditions [100]. Additionally, phosphate-deficient *Arabidopsis* plants exhibit hyperbolic coumarin and oligolignol exudation [101], and coumarin exudation is significantly reduced in ABCG37-deficient plants [102], serious metal-treated poplar (*Populus tremula*) iatrogenic organic acid exudation, and zinc-deficient wheat hyperbolic phytosiderophore exudation [103]. Differential exudation could be a plausible mechanism that plants might modulate their interaction with microbes. All of this evidence suggests that under different stresses the root secretions of plants are altered, and it is not difficult to speculate that the microbial community is altered accordingly. However, research on the secretion mechanism of plant inter-root secretions and the discovery of key loci for the recruitment of microorganisms remains a challenge.

## 3. Ecotype-Prone Splicing Events Are Good Candidates to Study Plant-Microbe Interactions

### 3.1. Pre-mRNA Alternative Splicing and Splicing Regulators of Plant Immunity

Most eukaryotic genes are interrupted by introns. Therefore, an important step in gene expression is the removal of introns through the splicing of precursor mRNA transcripts (premRNAs). Emerging evidence has indicated that plant-associated microbiomes are engaged with plant health and the helpful features of plant-associated microbes will boost the immune responses in plants against biotic/abiotic environmental constraints. Recent progress in high outturn sequencing of ribonucleic acid and bioinformatics tools to research AS events [104,105,106,107] at a genome-wide scale have shown that AS is a vital part of host transcriptome reprogramming in response to microorganism and viral infection in several plant species. In plants, varied reports have primarily focused on AS analysis in model plant species or nonwoody plants, resulting in a notable lack of research on AS in woody plants [108]. Alternative splicing (AS) enhances transcriptome malleability and proteome diversity in response to various growth and stress cues, which places AS at the crossroads of adaptation and environmental stress response [109,110]. However, some people are also working on different ways to improve these bottlenecks regarding AS. For example, Chen et al. presented the plant splicing-related protein expression and annotation database PlantSPEAD, introducing the information on their annotations, sequence information, functional domains, protein interaction partners, and expression patterns in response to abiotic stresses, from a dozen existing databases and the literature [111]. Furthermore, the SWATH-MS approach is characterized by a data-independent acquisition (DIA) method followed by a unique targeted information extraction approach [112]. SWATH-MS proteomics is applied for a range of large-scale profiling studies in plants, moving from model plant species to various plants without reference ordination annotation [113]. Within the field of discovery proteomics, alternative splicing is an emerging research area associated with posttranscriptional regulation. SWATH-MS could be applied to specifically establish peptides translated from splicing junctions [114]. This is also a direction worthy of further study in the future.

Plant immune receptors belonging to the receptor-like kinase (RLK) family play important roles in the recognition of microbial pathogens and activation of downstream defence responses. The function of RLKs in plant immune responses involves specific phosphorylation events within and outside the structural domain of the kinase, which leads to altered kinase activity and consequently to immune signalling. The function of several RLKs in pathogen resistance has been extensively studied in model plants with simpler genomes (e.g., rice and *Arabidopsis*). Examples include XA21 from rice [115,116] and PR5K from *Arabidopsis* [117]. The contribution of resistance to powdery mildew infection in durum wheat has been found by studying the expression and role of TtdLRK10 L-1 in wheat defence against powdery mildew infection and by identifying the intron putative MYB binding site (MYB-BS) in the positive role in the expression and function of TtdLRK10 L-1. Nucleotide binding site/leucine-rich repeat (NBS-LRR) and serine/threonine kinase (STK) genes are unit two of the known categories of resistance (R-) genes in plants and occur in massive multigene families. Some receptor-like kinases (RLKs) with serine/threonine were similar to both cytoplasmic RLKs, such as Pto, and RLKs with LRR, S-locus, lectin-like, and thaumatin-like extracellular binding-domains [118]. By analysing kinase domain-targeted isolation of defence-related receptor-like kinases (RLK/Pelle) in *Platanus* × *acerifolia*: phyletic and structural, we see that some RLKs have indeed been involved in the expression of phenotypic plasticity and are therefore a decent candidate for investigations into pathogen resistance [119].

PremRNA splicing plays an important role in the regulation of plant immunity mediated by the RLKs SNC4 and CERK1. Plant surface pattern receptors are variably spliced to produce different transcripts that respond to pathogen infestation by influencing downstream signalling. Bacterial flagellin 2 (FLS2) is a classical PRR receptor protein that detects conserved bacterial flagellin 2 (FLS2) in plants, sensing external bacterial infestation. Flagellin perception in Arabidopsis works through recognition of its extremely conserved N-terminal epitope (flg22) by flagellin-sensitive 2 (FLS2) to initiate a series of immune responses. In nine families of dicotyledons, FLS2 was shown to undergo AS of its first exon. Point mutations and gene swaps indicated that the position and potency of exitron splicing primarily relied on the nucleotide sequences of FLS2 genes. The position and efficiency of splicing depend mainly on the FLS2 nucleotide sequence of FLS2; exon of FLS2 is spliced via an intron-mediated enhancement (IME), which regulates the accumulation of transcriptional products. Some ATs have the potential to encode suppressors for the FLS2 pathway, and transformed transcripts can encode FLS2 pathway repressors that affect FLS2-mediated reactive oxygen species production. This study reveals that alternative splicing finely regulates the pattern of receptor recognition and thus influences the downstream disease resistance response [120]. The receptor-like cytoplasmic kinase BIK1, a component of the FLS2 immune receptor advanced, not solely positively regulates flg22-triggered calcium influx; it conjointly directly phosphorylates the NADPH oxidase RbohD at specific sites in an exceedingly calcium-independent manner to enhance ROS generation. However, at present, the relationship between some cytoplasmic kinases such as BIK1 and AS is unknown [121].

The *Arabidopsis thaliana* NPR1 loss-of-function mutant PR gene expression is reduced, and the ability to develop SAR loses the capacity to develop SAR. The backfill npr1 mutant to obtain the SNC (suppressor of npr1-1, constitutional) series of mutants can restore both abilities to some extent [122]. The *Arabidopsis thaliana* mutant SNC4 1D contains an RLK SNC4 (the NPR1-1 suppressor, CONSTITUTIVE4) gain-of-function mutation, which leads to constitutive activation of the defence response. Identification of two conserved shearing factors, SUA (ABI3-5 repressor gene) and RSN2 (SNC4-1D essential gene), are necessary to construct a defence response by mutating the snc4 1D mutant.

In SUA and RSN2 mutants, SNC4 shearing is altered, and further analysis showed that SUA and RSN2 are important receptors for shearing CERK1 (an essential receptor for PAMP, titin inducible receptor kinase 1). The precursor mRNA is sheared in the receptor-like kinase protein kinase SNC4 and CERK1-mediated regulation of plant immunity [123]. All these directly or indirectly express the key role played by alternative splicing in plant defence. Alternatively, spliced transcripts in plants result in profound changes in their gene expression patterns throughout developmental growth.

Interestingly, we found that AS events seem to have specificity across various plant species or in response to a variety of stressful environments. For resistance to different pathogenic bacteria, independent splice variants are also formed. IR was the most common AS pattern observed in *Paulownia tomentosa* [124]. Exon skipping was the primary AS pattern in *Populus* throughout salt stress, suggesting a completely different tree species produce different AS types to cope with constant stress. Apparently, an alternative acceptor is the major AS pattern when camellia sinensis is treated with drought and heat stress [125].

*Arabidopsis* RPS4 senses and is resistant to the Avr Rps4-expressing strain Pst.DC3000, and RPS4 undergoes variable splicing to produce six different transcripts. After infestation, RPS4 expression was upregulated in intact transcripts, whereas clonally incomplete transcripts could not backfill the resistance of the deficient RPS4 plants to *Pst. DC3000*, suggesting that only intact RPS4 transcripts are resistant to Pst [126]. In tobacco, the N gene specifically recognizes a 50 kD decapping enzyme protein (p50) of Tobaccomosaic virus (TMV) and selectively shears the N gene [127,128]. Different tree species manufacture completely different AS varieties to cope with a similar stresses. Curiously, an alternate acceptor is the major AS pattern when *C. sinensis* is treated with drought and heat stress [125]. Comparisons of gene structures from 67 plant species, protein domains, promoter regions, and conserved splicing patterns indicated that plant U1-70Ks are unable to preserve their preserved molecular function across plant lineages and play a very important practical role in response to environmental stresses [129]. A total of 4388 unique proteins were identified and quantified, among which 542 proteins showed vital abundance changes upon Pb(II) exposure, and differentially expressed proteins (DEPs) that were primarily distributed in the lignin and flavonoid synthesis pathways were powerfully activated upon Pb exposure, indicating their potential roles in Pb detoxification in poplar [130].

There are fewer reports related to alternative splicing, but it is easy to see that conserved AS events seem to be consistently present, including the feature that alternative splicing is specific under different stresses as well as under different species. We have also previously summarized the changes in microbial communities under different plants and different stresses, and it is unclear how the release of phytohormones relates to AS, including the recruitment of their preferred microbes by plants, so we speculate that AS may have its own unique splicing.

### 3.2. Genome-Wide Association Analyses of Splicing Quantitative Trait Loci (sQTLs) in Plants

Although AS is pervasive, the genetic basis for differential isoform usage in plants is increasing, widespread natural variation in AS has been observed in plants, and how AS is regulated and contributes to phenotypic variation is poorly understood. One study performed genome-wide analysis in 666 geographically distributed diverse ecotypes of *Arabidopsis thaliana* to spot genomic regions (splicing quantitative trait loci (sQTLs)) that regulated differential AS, and observed enrichment for trans-sQTLs (trans-sQTL hotspots) on chromosome regions. Many sQTLs were enriched, including the circadian clock, flowering, and stress-responsive genes, suggesting the potential role of differential isoform usage in controlling these necessary processes among diverse ecotypes of *Arabidopsis* [15]. The presence of widespread variation in diverse ecotypes of Arabidopsis at genetic level has been widely reported. For example, epithiospecifier protein (ESP) is responsible for diverting glucosinolate hydrolysis from the generation of isothiocyanates to that of epithionitriles or nitriles and thereby negatively affecting the capacity of the plant to defend itself against certain insects. Some studies have shown that ESP expression is regulated differently between the two *A. thaliana* ecotypes [131]. Comparison of AS in three phenotypic variants of maize revealed the importance of AS in diversifying gene function and regulating phenotypic variation as well [132] (Table 1).

### 3.3. Crosstalk between Plant Ecotype-Specific Splicing and Their Commensal Microbiome

**Figure 1:**
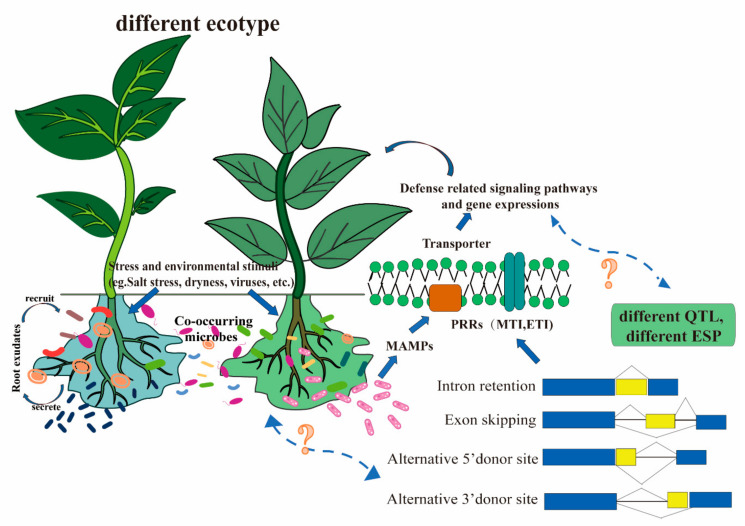
Summary model and research gaps among plant ecotypes, commensal microbiota, and alternative splicing-associated quantitative trait loci. Ecotype-specific microbial recruitment, and hypothesized relationship between plant induced alternatively spliced genes. Different plants, different genotypes, and different stresses will all release different root secretions and recruit microorganisms according to their needs to build their own unique communities in response to certain biotic or abiotic stresses. There is much evidence that PRRs in many plants are associated with AS. However, with different stresses, different plants have different preferences for AS, and different ecotypes of plants have differences in sQTLs that are enriched in flowering and stress response genes. Therefore, we speculate that alternative splicing is somehow associated with expression shape regulation and microbial recruitment, and plant-induced gene expression.

Increasing evidence has suggested that the splice sites were also differentially selected among various plant ecotypes. Although the effect of genotypic variation on splice site determination among plant ecotypes is less reported, the sQTLs identified in several studies provide fundamental evidence to support this finding. Subsequently, an intriguing hypothesis has been proposed that the splicing variation among plant ecotypes may further influence the recruitment of the plant commensal microbiome by affecting crucial factors involved in secondary metabolism, root exudate secretion, and plant immune responses, etc. However, the role of these differential splice sites present among various plant ecotypes during the interaction of plants and their microbiomes remains unclear (Figure 1).

## 4. Conclusions and Future Perspective

In recent years, the study of plant microbiome interactions has become a hotspot in scientific research. Increasing evidence suggested that alternative splicing plays a pivotal role in plant–microbiome interactions, a dynamic process controlled by both factors generated by plants and microorganisms. Specifically, in this review article, we discussed the recruitment mechanism of commensal microbiome by plant hosts and proposed that the differential recruitment of the microbiome observed among plant ecotypes is closely linked to their splicing quantitative trait loci (sQTLs) identified from these plant ecotypes. For instance, a deep understanding of the genetic linkage between AS and secretion of root exudates for microbiome recruitment will be the key to mastering changes in plant ecology and plant breeding. However, further investigations are required to validate this hypothesis and proof of its value to form the new research direction in this field.

To this end, studies of both host and microbiome aspects are necessary to unravel the underlying mechanism between plant–microbiome interactions. In the host aspect particularly, the propensity for AS varies under different stress conditions, and unique splice variants are developed to fight against different pathogens. AS triggers intracellular defence signals and mass expression of defence genes, yet the interactions between different plant-associated symbionts and the plant immune system are unknown. Plants recruit microbes in a ‘call for help’ strategy, with different species recruiting different microbes and susceptible versus resistant species recruiting different microbial communities. One major challenge is going to be to research root exudation in natural settings. Due to the chemical complexity of soil, exudation is historically analysed in aquacultural culture, an environment distant from many natural settings of plant microbiome studies. In addition, new technologies are needed for high-throughput screening of functional microorganisms that can be targeted to reveal the impact of core microorganisms on the inter-root biota and, in turn, on plant health. With further understanding of root morphology and secretions, the core strains involved may alter the engineering or breeding of plants with altered talent to act against pathogens. This must be complemented with an improved understanding of the substrate preferences of plant-associated microbes, their interactions, and also the mechanisms which profit the plant. Although research on alternative splicing in plant disease resistance has been ongoing, there are still many unanswered questions about the role of alternative splicing in plant immunity.

Regarding commensal microbes, with the evolution of host–microbe interactions, microorganisms have evolved evolutionary strategies for manipulating host AS machinery to disrupt host immunity, and alternative splicing is involved in the mechanism of R-Avr interactions. Whether this mechanism is widespread in multiple species and in disease-resistance systems in which different microorganisms interact with plants has not been fully explained. The mechanism of alternative splicing in the R–Avr interaction, and whether it is widespread in multiple species and in different microbe–plant interactions in the disease resistance system, is also not fully understood; nor are the functional mechanisms of R proteins and subcellular localization. In the future, we need to investigate the functional mechanism, subcellular localization, and interaction with the target protein Avr to further expand our understanding of the molecular mechanism of plant resistance.

## Figures and Tables

**Table 1:** A comparison of some representative microbial communities of different genotypes and a comparison of alternative splicing of plants of different ecotypes is summarized.

Plant Varieties	Genotype Varieties	Region	Reproductive Stages	Result
Wheat	Apache (AP), Bermude (BM), Carstens (CT), Champlein (CH), Cheyenne (CY), Rubisko (RB), Soissons (SS), Terminillo ™	Senegal, Cameroon, France, and Italy	Harvest season	Plant genotypes and soil have specific effects on the wheat rhizosphere microbial community.
Rice	Zhefu No. 7, hereafter: HA, Xiangzaoxian No. 45, hereafter: LA	Zhejiang Province, China	Harvest season	Significant differences in the composition of inter-root bacterial communities of different rice varieties.
Soybean	Kwangan (KA), Poongsannamul (PS), Poongwon (PW), and Taekwang (TK)	Jeonju, South Korea	R1, R3, R5, and R7	Influenced by the variety and growth stage.
Winter Wheat	Eltan, Finch, Hill81,Lewjain, Madsen, PI561722, PI561725, PI561726 and PI561727	Pullman, WA, USA.	Late May to early June, 2010 and 2014	Wheat cultivars are involved in shaping the rhizosphere by differentially altering the bacterial OTUs consistently across different sites.
Maize	Zhengdan 958 (ZD), Gaoneng 1(G1), and Gaoneng 2 (G2)	Hebei province, China	18 September 2017	The bacterial community structure in bulk soil of different cultivars was significantly different.
Rabbiteye blueberry	Clearly clustered into three groups (BRI, BRII, and RBIII).	Lishui, Nanjing province, China	Harvest Season	The rhizosphere from the plant cultivars exerted substantial effects on bacterial association networks and putative keystone species.
Tomato	Resistant varieties and non-resistant varieties	Seoul, Republic of Korea.	Harvest season	More abundant in the resistant plant rhizosphere microbiome than in that of the susceptible plant.
Bean	Resistant varieties and non-resistant varieties	Anhembi municipality, Sao Paulo, Brazil	Harvest season	Different levels of resistance to the fungal root pathogen Fusarium (Fox), differences in microbial composition.
Plant Varieties	Genotype Varieties	Unique Splicing quantitative trait loci (sQTL)	Reproductive Stages	Result
*Arabidopsis thaliana*	666 geographically distributed diverse ecotypes	6406	/	A role for differential isoform usage in regulating these important processes in diverse ecotypes of *Arabidopsis*.
Maize	Maize kernels from 368 inbred lines	19,554	/	The importance of AS in diversifying gene function and regulating phenotypic variation.

## Data Availability

Not applicable.
